# Optimization of Dosage for Asphalt Volatile Harmful Gas Inhibitor Using Multi-Response Satisfaction Function and Nonlinear Regression

**DOI:** 10.3390/ma19091871

**Published:** 2026-05-01

**Authors:** Zhiye Liu, Xiaoyu Ren, Wenyao Du, Qinghang Li, Dedong Guo, Meng Xu, Wei Lu, Chiara Riccardi, Mengchen Li, Zouwei Zhong

**Affiliations:** 1 Shandong Key Laboratory of Technologies and Systems for Intelligent Construction Equipment, Shandong Jiaotong University, Jinan 250357, China; 24107025@stu.sdjtu.edu.cn (Z.L.); guodedong@sdjtu.edu.cn (D.G.); 24107017@stu.sdjtu.edu.cn (M.L.); 24107055@stu.sdjtu.edu.cn (Z.Z.); 2 School of Highway and Construction Engineering, Yunnan Communications Vocational and Technical College, Kunming 650500, China; 2025030302@ynjtc.edu.cn; 3 Jining Jizou Expressway Co., Ltd., Jining 272067, China; d26100920@163.com; 4 Shandong Pengcheng Road and Bridge Group Co., Ltd., Jining 272499, China; z225971@163.com; 5 Jinan Urban Construction Group, Jinan 250031, China; 6 Department of Civil and Industrial Engineering, University of Pisa, Largo Lucio Lazzarino 1, 56126 Pisa, Italy; chiara.riccardi@unipi.it

**Keywords:** multi-asphalt system, volatile harmful gases from asphalt, composite inhibitor, nonlinear regression, satisfaction function, multi-objective optimization

## Abstract

To achieve synergistic, efficient degradation of volatile, harmful gases in asphalt and to scientifically quantify inhibitor dosage, this study proposes a dosage optimization method that integrates nonlinear regression with a multi-response satisfaction function. Focusing on a proprietary composite volatile gas suppressant, we systematically measured the concentration trends of ammonia, nitrogen oxides, sulfur dioxide, and hydrogen sulfide emitted from three asphalt systems: base asphalt, SBS modified asphalt (Styrene-Butadiene-Styrene modified asphalt), and rubber modified asphalt under different suppressant dosages (0%, 0.02%, 0.04%, 0.06%, 0.08%, and 0.10%). First, high-precision prediction models (*R*^2^ > 0.95) were established using nonlinear regression to relate different inhibitor dosages to corresponding gas concentrations. Based on a satisfaction function, the multi-objective degradation effects were normalized into a comprehensive satisfaction index, and the optimal dosage was then determined. The results indicate: (1) the constructed models can accurately predict the concentrations of volatile harmful gases at various dosages; (2) the predicted optimal blending ratios vary by asphalt type, specifically 0.082% for base asphalt, 0.079% for SBS modified asphalt, and 0.080% for rubber modified asphalt; and (3) at the optimal blending ratios, all four gases achieve high and balanced degradation levels, resulting in the best overall degradation performance. At the same time, road performance tests confirmed that this blending ratio has no significant negative impact on the high-temperature and low-temperature stability or water stability of the asphalt mixture. Compared with traditional single-factor empirical methods, this approach represents a methodological upgrade from qualitative description to quantitative prediction, and from single-objective comparison to multi-objective synergistic optimization, providing data and theoretical support for the precise, efficient, and engineering-applicable use of asphalt volatile gas inhibitors.

## 1. Introduction

Asphalt fumes released during the production and construction of hot-mix asphalt contain various volatile organic compounds and irritating harmful gases, severely polluting the environment and endangering the health of construction workers [1,2,3]. Adding volatile gas inhibitors is one effective method to suppress harmful gas emissions at the source. Current research primarily focuses on developing novel inhibitors [4,5,6,7] and verifying their degradation effects [8,9,10]. However, current research has not yet achieved a balance between the degree of degradation of harmful fumes generated during the mixing of asphalt mixtures and their road performance. It thus cannot provide effective guidance on the appropriate dosage of inhibitors. When determining optimal dosage levels, single-factor experiments are commonly employed. This involves comparing trends in single or multiple gas concentrations across different dosages and establishing a dosage range based on engineering experience. While this method is simple and intuitive, it has significant limitations: First, it relies on discrete experimental data points for trend analysis, lacking quantitative models capable of continuous prediction [11]. Second, when dealing with multiple harmful gases that need to be controlled simultaneously, the optimal concentration ranges for achieving the best degradation efficiency may not overlap [12]. Simple observational methods make it difficult to scientifically weigh the merits of multiple targets and cannot accurately determine the optimal concentration.

Therefore, there is an urgent need to introduce more systematic and precise mathematical methods to advance the optimization of inhibitor dosages. Response surface methodology [13,14] is a powerful tool for solving multi-factor, multi-response optimization problems, but its full application typically requires at least two continuous independent variables. When the core objective is to optimize a single factor (dosage), combining nonlinear regression with multi-response optimization algorithms provides an efficient and accurate alternative. Therefore, compared to traditional empirical methods (such as single-factor equal-step trial-and-error methods or equal-weighting methods), the use of nonlinear regression combined with a multi-response satisfaction function can more accurately describe continuous dose–response relationships and provide optimal dosage decisions when balancing the trade-offs among the degradation effects of various gases. Nonlinear regression establishes precise quantitative relationship models between variables. At the same time, multi-response optimization algorithms (such as the satisfaction function method [15]) integrate multiple competing or correlated objectives into a single comprehensive metric for optimization.

Based on this, this paper takes a composite inhibitor independently developed by our team as an example. First, experimental data were obtained on how the concentrations of four major harmful gases (NH_3_, NO_x_, SO_2_, and H_2_S) in three typical asphalt systems vary with the amount of inhibitor added. Subsequently, a high-precision nonlinear prediction model was established to address the technical challenges involved in continuous quantitative prediction. Finally, using the satisfaction function method, the degradation effects of the four gases were comprehensively and synergistically optimized to determine the precise dosage that maximizes overall satisfaction. This study aims to achieve the following objectives: (1) achieve a comprehensive satisfaction rate of over 85% in each asphalt system; (2) ensure that the degradation rates of all four harmful gases are no less than 40%; and (3) predict the inhibitor dosage as accurately as possible to provide clear dosage guidelines for engineering applications. This study aims to develop precise, quantitative, and scientifically rigorous guidelines for the application of inhibitors to minimize emissions of harmful asphalt fumes without compromising the performance of asphalt pavements, thereby advancing the development of asphalt pavement materials toward greater environmental sustainability and precision [16,17].

## 2. Materials and Test Methods

### 2.1. Materials

(1) Base asphalt

This study used Qilu brand 70# asphalt (Shandong Hi-Speed New Materials Group Co., Ltd., Jinan, China) as the base asphalt. It was tested in accordance with the “Standard Test Methods for Asphalt and Asphalt Mixtures in Highway Engineering” (JTG 3410–2025) [18], and all technical indicators met the relevant requirements of the “Technical Specifications for Construction of Highway Asphalt Pavements” (JTG F40-2004) [19]. The test results are shown in Table 1.

(2) SBS modified asphalt

The SBS modified asphalt selected for this study is Type I-D SBS modified asphalt supplied by Shandong Hi-Speed New Materials Group Co., Ltd. (Jinan, China). As shown in Table 2, all performance indicators meet the requirements of the Technical Specifications for Construction of Highway Asphalt Pavements (JTG F40-2004) [19].

(3) Rubber modified asphalt

The rubber modified asphalt selected for this study is rubber powder modified asphalt supplied by Shandong Hi-Speed New Materials Group Co., Ltd. (Jinan, China). As shown in Table 3, all of its performance indicators meet the requirements of the Technical Standard for Rubberized Asphalt Pavement (CJJ/T 273-2019) [23].

(4) Inhibitor

The inhibitor used in this study is a composite asphalt fume suppressant developed in-house by our research group. It is a pale yellow, transparent liquid with a density (at 25 °C) of 0.95 g/cm^3^, a flash point (open cup) of 178 °C, and a neutral pH. Adding this inhibitor during the production of hot-mixed asphalt mixtures effectively removes harmful volatile gases from the asphalt.

(5) Mineral aggregate and mix proportion

This study employed limestone coarse aggregate, fine aggregate, and limestone powder, with key technical indicators including crush value, abrasion value, bulk density, water absorption rate, sand equivalent, and hydrophilic coefficient, all meeting the relevant requirements for mineral aggregates specified in the Technical Specifications for Construction of Highway Asphalt Pavements (JTG F40-2004) [19]. The mixture gradation adopts the AC-13 type, with a synthetic gradation designed based on the standard median value. The pass rates for all key sieve openings fall within the specified requirements.

Using the Marshall test method, the optimal binder-to-aggregate ratios for 70# asphalt, SBS modified asphalt, and rubber modified asphalt mixtures were determined to be 4.8%, 5.1%, and 6.2%, respectively, and these ratios were used for the uniform preparation of all subsequent test specimens.

### 2.2. Sample Preparation and Gas Collection Detection

Heat the asphalt to the specified temperature (160 °C for base asphalt, 180 °C for modified asphalt). Place the asphalt with different admixture dosages (0%, 0.02%, 0.04%, 0.06%, 0.08%, 0.10%) in a high-speed shear mixer. The mixture is pre-mixed at 1200 r/min for 5 min, followed by high-speed shearing at 4000 r/min for 25 min to produce a homogeneous sample [24]. The optimum binder-to-aggregate ratio for three asphalt mixtures was determined using the AC-13 gradation and Marshall design method, and specimens were molded.

**Figure 1:**
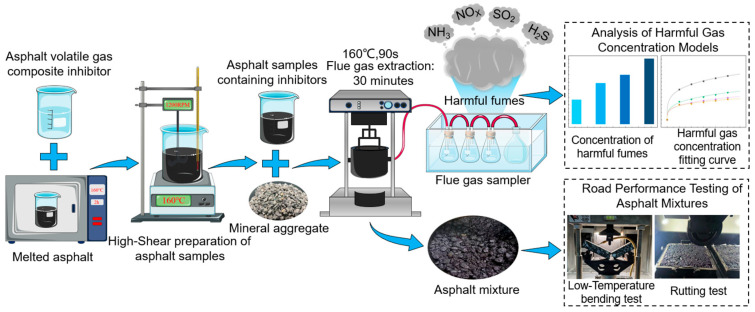
Harmful fumes simulation collection device.

A laboratory simulation apparatus (see Figure 1) was employed to collect gases released from asphalt mixtures during high-temperature mixing [25]. A specified mass of mixture (12,000 g) was heated and mixed in a sealed mixing pot. The generated gases were drawn through a conduit by a vacuum pump into a series-connected flue gas sampler (Ly-3072 Intelligent Dual-Channel Flue Gas Sampler; Qingdao Laoshan Applied Technology Research Institute, Qingdao, China). Ammonia (NH_3_), nitrogen oxides (NO_x_, expressed as NO_2_), and sulfur dioxide (SO_2_) were collected using dilute sulfuric acid absorption solution, hydrochloric acid-naphthylenediamine absorption solution, and formaldehyde buffer absorption solution, respectively. Hydrogen sulfide (H_2_S) is collected in gas sampling bags and absorbed using a cadmium hydroxide-polyvinyl alcohol ammonium phosphate solution [26]. (1) For NH_3_, a diluted sulfuric acid solution was used as the absorption solution. According to HJ 533-2009 [27] (Ambient air and exhaust gas—Determination of ammonia—Nessler’s reagent spectrophotometry), the absorption solution is 0.05 mol/L sulfuric acid(H_2_SO_4_). (2) For NO_x_(calculated as NO_2_), the absorption solution was prepared according to HJ 479-2009 [28] (Ambient air—Determination of nitrogen oxides—N-(1-Naphthyl) ethylenediamine dihydrochloride spectrophotometric method). The absorption solution typically consists of glacial acetic acid, sulfanilic acid, and N-(1-Naphthyl) ethylenediamine dihydrochloride. (3) For SO_2_, a formaldehyde buffer absorption solution was used. According to HJ 482-2009 [29] (Ambient air—Determination of sulfur dioxide—Formaldehyde absorption—pararosaniline spectrophotometry), the formaldehyde buffer stock solution is diluted 100 times with pure water before use to prepare the formaldehyde buffer absorption solution. (4) For H_2_S, an absorption solution containing cadmium hydroxide-polyvinyl alcohol-ammonium phosphate was used. According to the methylene blue spectrophotometric method specified in the fourth edition of the “Air and Exhaust Gas Monitoring and Analysis Methods”, the absorption solution is prepared by dissolving 4.3 g of cadmium sulfate (3CdSO_4_·8H_2_O), 0.3 g of sodium hydroxide (NaOH), and 10 g of polyvinyl alcohol-ammonium phosphate in water, mixing thoroughly, and diluting to 1000 mL, resulting in a milky white suspension. All gas concentrations are measured using a UV-visible spectrophotometer (Qingdao Laoshan Applied Technology Research Institute, Qingdao, China) in accordance with the corresponding national environmental protection standard methods [30]. Finally, the collected data were organized using Origin 2024 software.

### 2.3. Road Performance Testing of Asphalt Mixtures

To evaluate the effect of composite volatile gas inhibitors on the basic in-service performance of asphalt mixtures, test specimens were prepared for each type of asphalt at the optimal dosage determined through optimization and compared with blank samples (0% dosage) [31,32,33,34]. In accordance with the “Test Procedures for Asphalt and Asphalt Mixtures in Highway Engineering” (JTG 3410-2025) [18], as shown in Table 4, the key in-service performance tests were conducted, with each test performed in at least three parallel runs.

### 2.4. Principles and Process of Multi-Response Optimization

#### 2.4.1. Establishing Nonlinear Regression Models

For each type of asphalt, a mathematical model was established to describe the relationship between the concentrations of four harmful gases (y) and the inhibitor content (x) [35]. In this study, quadratic and cubic fitting curves were used to construct a nonlinear regression model, which effectively captures the trend where gas concentrations first decrease rapidly and then stabilize as the inhibitor dosage increases. This is consistent with classical adsorption theory: at low concentrations, the inhibitor provides sufficient active sites, resulting in high degradation efficiency; as the concentration continues to increase, the active sites are gradually occupied by harmful gas molecules, leading to a decrease in degradation efficiency and a flatter curve. The specific model forms are as follows:

(1) $$\text{Second-order}\;\mathrm{polynomial}\;\text{model:}\;y=\beta_{0}+\beta_{1}x+\beta_{2}x^{2}+\epsilon$$

(2) $$\text{Three-fold}\;\text{multi-form}\;\text{model:}\;y=\beta_{0}+\beta_{1}x+\beta_{2}x^{2}+\beta_{3}x^{3}+\epsilon$$

Among these, $\beta_{i}$ represents the regression coefficient, and ϵ denotes the random error. Based primarily on the coefficient of determination (*R*^2^) and residual analysis, the model with the best goodness-of-fit is selected.

#### 2.4.2. Satisfaction Function Method

To simultaneously optimize the degradation efficiency of four harmful gases (NH_3_, NO_2_, SO_2_, H_2_S), the satisfaction function method proposed by Derringer and Suich [36] was adopted. This method converts each response ($y_{i}$) into an individual satisfaction value ($d_{i}$) ranging from 0 to 1.

(1) Individual Satisfaction ($d_{i}$)

For harmful gas concentrations, the goal is “the lower, the better.” A linear decreasing function is used for conversion:

(3) $$d_{i}=\left\{\begin{array}{cc} 1, & y_{i}\leq y_{i}^{min}\\ \frac{y_{i}^{max}-y_{i}}{y_{i}^{max}-y_{i}^{min}}, & y_{i}^{min}<y_{i}<y_{i}^{max}\\ 0, & y_{i}\geq y_{i}^{max} \end{array}\right.$$

Among these, $y_{i}^{min}$ and $y_{i}^{max}$ represent the minimum and maximum concentrations observed for the i-th gas within the experimental dilution range (0–0.10%), respectively.

(2) Overall Satisfaction (*D*)

The geometric mean of the satisfaction scores for each gas is calculated to obtain the overall comprehensive satisfaction score *D*, which serves as the final objective function for multi-response optimization.

(4) $$D=(d_{1}\times d_{2}\times d_{3}\times d_{4}{)}^{1/4}$$

The closer the overall satisfaction score *D* is to 1, the more ideal the overall degradation effect of the four gases.

#### 2.4.3. Optimize the Solution Process

(1) Utilize the established nonlinear regression model to predict gas concentrations at densely spaced blending points (intervals of 0.001%).
(2) Calculate the individual satisfaction ($d_{i}$) for each gas corresponding to each blending point based on the predicted concentrations and the maximum/minimum values within the experimental range.
(3) Calculate the comprehensive satisfaction (*D*) corresponding to each blending point.
(4) Identify the mixing ratio x* that maximizes the overall satisfaction *D*, which represents the theoretical optimum mixing ratio for that asphalt type.

## 3. Results and Discussion

### 3.1. Establishment and Validation of Nonlinear Regression Models

**Figure 2:**
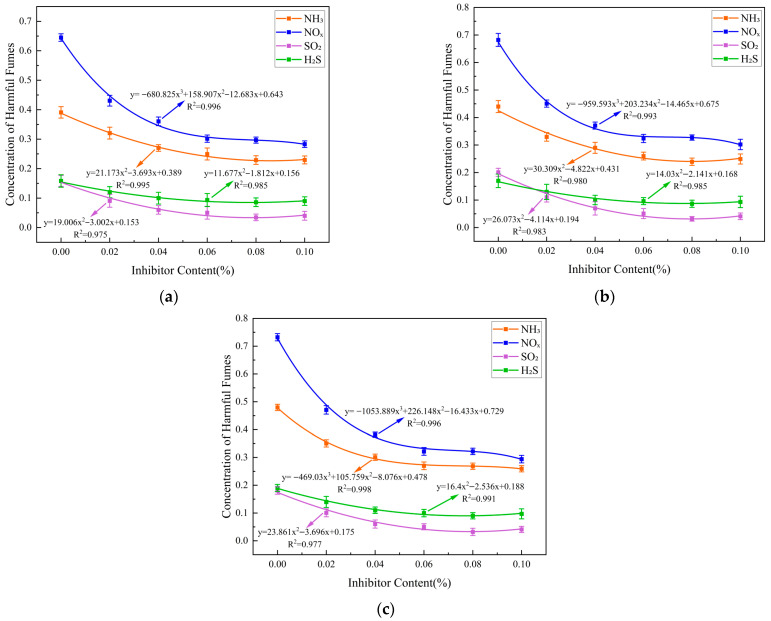
Concentrations of harmful fumes versus inhibitor content and fitting curves for three types of asphalt: (**a**) 70# asphalt, (**b**) SBS modified asphalt and (**c**) rubber modified asphalt.

Scatter plots were plotted for the concentrations of four gases collected by the harmful fume simulation collection device, showing the relationship between the concentrations of harmful fumes released by three asphalt systems and the inhibitor dosage. Nonlinear curve fitting was performed using quadratic or cubic polynomials, with the fitted curves shown in Figure 2 below.

A significance analysis was performed on the fitting results for the three asphalt systems shown in the figure above, and the results are summarized in Table 5. All regression models passed the F-test (*p* < 0.01), indicating that the nonlinear relationship between inhibitor dosage and gas concentration is statistically significant. Furthermore, the coefficient of determination (*R*^2^) for all fitted curves was greater than 0.95, indicating high fitting accuracy and suggesting that these equations can be used to predict gas concentration values for the three asphalt systems at different inhibitor ratios. To verify the model accuracy, we conducted a repeat test using a 0.05% additive concentration; the deviation between the measured values and the model predictions did not exceed 5%, ruling out the possibility of overfitting.

**Figure 3:**
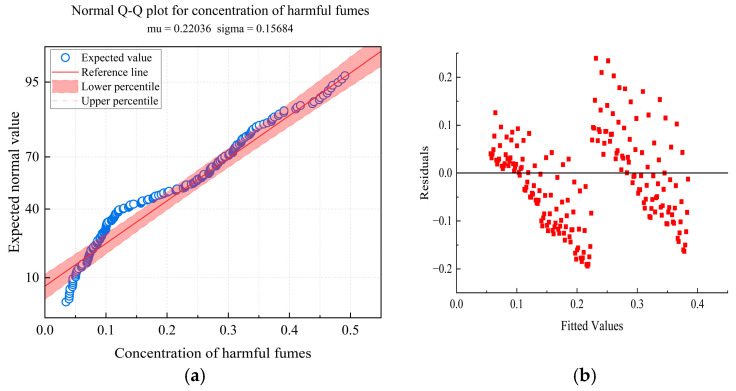
(**a**) Normal Q-Q plot for concentration of harmful fumes. (**b**) Residuals vs. fitted values.

To ensure the reliability of the analysis of variance results, residual diagnostics were conducted. As shown in Figure 3, the standard Q-Q plot for the degree of blending reveals points distributed along the diagonal, indicating that the normality assumption is mainly satisfied. The residuals versus fitted values plot, where the points are randomly scattered around the zero line with no discernible pattern, supports the assumption of homoscedasticity. As experiments were conducted in random order, the independence assumption holds. In summary, the model assumptions are reasonably satisfied, rendering the aforementioned statistical inference reliable.

The models used to fit the emission curves of harmful fumes from the three different asphalt systems all demonstrated strong explanatory power regarding data variability and yielded excellent fitting results, thereby validating the models’ applicability. These high-precision models lay a reliable foundation for subsequent prediction and optimization.

### 3.2. Comprehensive Optimization Analysis Based on Satisfaction Functions

Using the model obtained from the fitted curve, the gas concentration values were predicted for each asphalt system with inhibitor content ranging from 0% to 0.10% (in increments of 0.001%). Subsequently, the individual satisfaction ($d_{i}$) and overall satisfaction (*D*) corresponding to each dosage point were calculated based on Equations (3) and (4).

**Figure 4:**
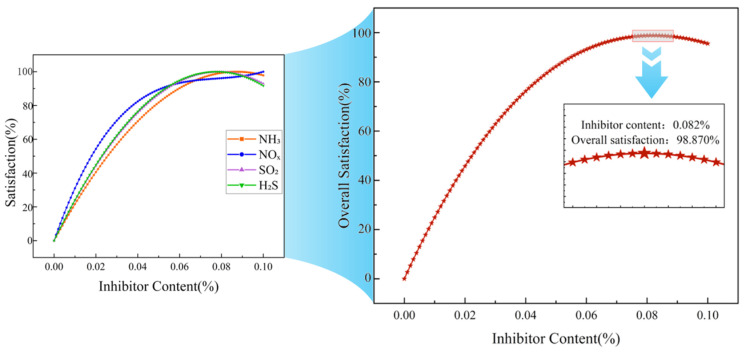
Individual and overall satisfaction curves for harmful fumes from 70# asphalt.

**Figure 5:**
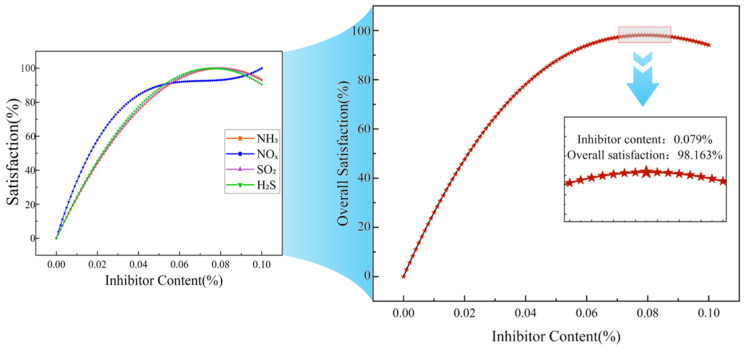
Individual and overall satisfaction curves for harmful fumes from SBS modified asphalt.

**Figure 6:**
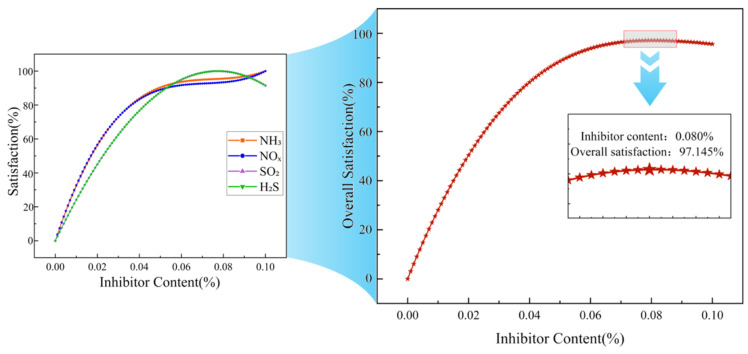
Individual and overall satisfaction curves for harmful fumes from rubber modified asphalt.

Figure 4, Figure 5 and Figure 6 show the satisfaction change curves for the 70# asphalt, SBS modified asphalt, and rubber modified asphalt systems, respectively.

A comparison of Figure 4, Figure 5 and Figure 6 shows that the trends in individual satisfaction levels for various gases across the three asphalt systems are generally consistent with changes in inhibitor dosage, differing only slightly in specific values.

Regarding NO_x_, the individual suppression efficiency increased most rapidly in the 70# asphalt and SBS modified asphalt systems, reaching 80% at inhibitor dosages of approximately 0.037% and 0.036%, respectively. Subsequently, the increase slowed, with both systems reaching maximum individual suppression efficiency at a dosage of 0.10%. In the rubber modified asphalt system, the rates of increase for NO_x_ and NH_3_ were comparable, reaching 80% at an inhibitor dosage of 0.037% and peaking at 0.10%.

The individual satisfaction curves for H_2_S and SO_2_ were relatively similar across all systems. In the 70# asphalt system, both reached 80% at inhibitor dosages of 0.043% and 0.044%, respectively, and reached maximum individual satisfaction at 0.078% and 0.079%. In the SBS modified asphalt system, both reached 80% at inhibitor dosages of 0.043% and 0.044%, respectively, and reached maximum individual satisfaction at 0.076% and 0.079%. In the rubber modified asphalt system, both reached 80% at an inhibitor dosage of 0.043%, and simultaneously reached maximum individual satisfaction at 0.077%.

NH_3_ exhibited the slowest increase in individual satisfaction across all systems. In the 70# asphalt system, it reached 80% at an inhibitor dosage of 0.049% and achieved maximum individual satisfaction at 0.087%; in the SBS modified asphalt system, it reached 80% at an inhibitor dosage of 0.044% and peaked at 0.080%; in the rubber modified asphalt system, it reached 80% at an inhibitor dosage of 0.036% and peaked at 0.10%.

For the four harmful gases in the three asphalt systems, individual satisfaction levels all show an upward trend as the additive dosage increases. However, the rate of increase and the “inflection points” differ. A comparison of the satisfaction curves across the three asphalt systems reveals that different gases exhibit varying sensitivities to the inhibitor [37], and the effects of the inhibitor on different asphalt systems also differ [38].

### 3.3. Comparison of Optimal Blending Ratios for Three Types of Asphalt

Through optimization calculations, the optimal inhibitor content for maximizing the comprehensive satisfaction of three types of asphalt was determined. The results are summarized in Table 6.

**Figure 7:**
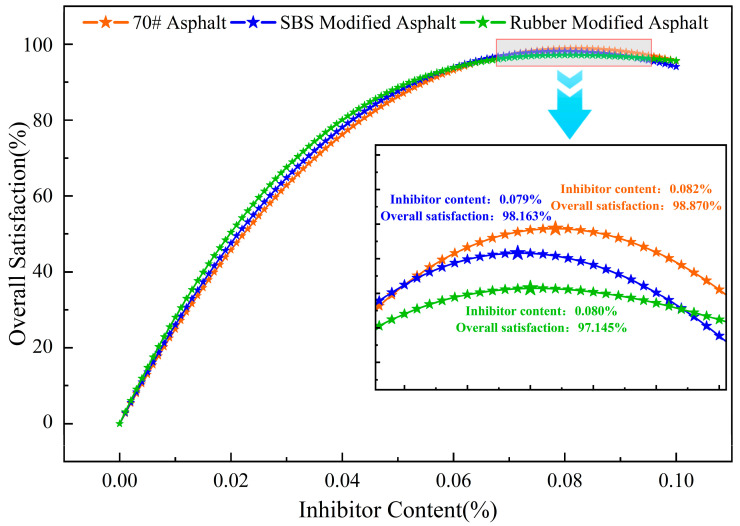
Comparison of overall satisfaction curves for three types of asphalt.

Figure 7 shows a comparison of the overall satisfaction curves for the three types of asphalt. It can be seen that, as the inhibitor dosage varies, all curves exhibit a single peak. The overall satisfaction of the 70# asphalt system reached its maximum value (*D_Max_* ≈ 98.870%) at an inhibitor dosage of 0.082%. The SBS modified asphalt system achieved maximum overall satisfaction at an inhibitor dosage of 0.079% (*D_Max_* ≈ 98.163%); the rubber modified asphalt system reached maximum overall satisfaction at an inhibitor dosage of 0.080% (*D_Max_* ≈ 97.145%). These three inhibitor dosages represent the theoretically optimal levels achieved after balancing the degradation effects of all four gases. At those dosages, it is not that the degradation of any single gas is optimized, but rather that all four gases achieve a high and relatively balanced level of degradation (with individual satisfaction scores all above 0.85), thereby achieving the best overall results.

The peak heights and positions of the overall satisfaction curves for the three asphalt systems differ, reflecting variations in the effectiveness of the inhibitor across different asphalt systems. Since 70# asphalt contains the highest proportion of light fractions, which are more prone to forming smoke during heating [39], the 70# asphalt system actually requires a higher dosage of the inhibitor. In contrast, SBS modified asphalt forms a stable three-dimensional network structure within the asphalt matrix [40]. This structure enhances the viscoelasticity of the asphalt while effectively reducing the migration and volatilization of light fractions. Consequently, the inhibitor in this system can act more precisely on free polar molecules, achieving optimal results at the lowest dosage (0.079%). Although rubber modified asphalt has high viscosity, components such as carbon black in the rubber powder also adsorb harmful gases [41]. However, this adsorption may compete with the inhibitors [42], causing some of the active sites of the inhibitors to be physically masked. Consequently, its optimal dosage (0.080%) falls between those of SBS modified asphalt and 70# asphalt.

Using the model obtained from the fitted curve, the gas concentration values for the three asphalt systems were predicted at the optimal inhibitor dosage. The degradation rates of each gas at this optimal dosage were calculated for the three asphalt systems, and the results are summarized in Table 7.

**Figure 8:**
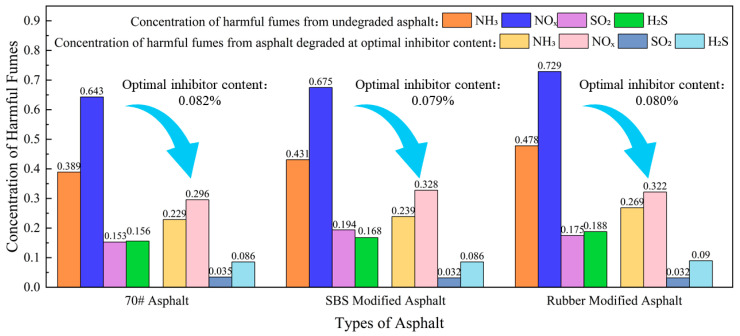
Comparison of harmful fume concentrations in asphalt before and after degradation.

Analysis of Figure 8 shows that this inhibitor significantly reduces the harmful gases produced by all three asphalt systems. However, when considering the optimal inhibitor dosage for all three asphalt systems, it was found that although the dosage for 70# asphalt was the highest, its final reduction in most gases was not significantly better than that of the other two asphalt systems. From the perspective of application economics and efficiency, SBS modified asphalt achieves optimal SO_2_ reduction at the lowest dosage. Rubber modified asphalt exhibits unique inhibitory advantages against H_2_S. For 70# asphalt, excessive dosage may not yield proportional efficiency gains. Overall, the inhibitor demonstrated higher removal efficiency for sulfur-containing compounds (SO_2_, H_2_S) than for nitrogen-containing compounds (NH_3_, NO_x_). This suggests the inhibitor’s mechanism of action may preferentially bind to sulfur-containing active sites within the asphalt or exhibit higher affinity for sulfur-based volatiles.

### 3.4. Analysis of the Influence of Optimal Asphalt Content on the Road Performance of Asphalt Mixtures

Table 8 lists the test results for the key in-service performance properties of the three asphalt mixtures at the optimized inhibitor dosage, along with the percentage change relative to the control group.

As shown in Table 8, at the optimal dosage, the addition of a composite volatile gas inhibitor ensures that all key performance indicators for all asphalt mixture types still meet the technical requirements of the Technical Specifications for Construction of Highway Asphalt Pavements (JTG F40-2004) [19]. Compared with the blank samples, the dynamic stability (*DS*) of 70# asphalt, SBS modified asphalt, and rubber modified asphalt incorporating the optimal inhibitor dosage decreased by 2.85%, 2.82%, and 2.46%, respectively. The freeze–thaw splitting resistance ratio (${TSR}_{f}$) increased by 0.78%, 0.88%, and 0.97%, respectively. At the same time, the maximum flexural strain $\varepsilon_{B}$ increased by 3.32%, 3.26%, and 3.12%, respectively. All performance indicator variation rates remained within ±5%, which is within the permissible error range of the test method. This indicates that while achieving efficient gas degradation, the inhibitor did not significantly adversely affect the high-temperature, low-temperature, or water stability properties of the asphalt mixture. This confirms the inhibitor’s excellent material compatibility and engineering applicability [43].

## 4. Conclusions

(1) An optimization method combining nonlinear regression with a multi-response satisfaction function was proposed, marking a methodological leap from empirical judgment to scientific quantification and multi-objective coordinated optimization of the dosage of asphalt volatile gas inhibitors.
(2) For three typical asphalt systems, high-precision prediction models (*p* < 0.01, *R*^2^ > 0.95) were established linking harmful smoke concentrations to suppressant dosage. The optimized dosages are: 0.082% for 70# asphalt, 0.079% for SBS modified asphalt, and 0.080% for rubber modified asphalt. At these dosages, gas degradation achieves a high and balanced overall optimum.
(3) Road performance tests demonstrated that at the optimized dosage, the inhibitor had no significant adverse effects on the high-temperature, low-temperature, or water stability of the asphalt mixture, confirming the integration of its environmental benefits with engineering applicability.

## Figures and Tables

**Table 1:** Physical properties of 70# asphalt.

Test Metrics	Technical Requirements	Test Results	Test Method [18]
Ductility (1 cm/min, 10 °C, cm)	≥20	29.3	T 0605-2011
Softening point (°C)	≥46	47.6	T 0606-2011
Penetration (25 °C, 100 g, 5 s, 0.1 mm)	60~80	72.1	T 0604-2011
Solubility (trichloroethylene, %)	≥99.5	99.6	T 0607-2025
Flash point (°C)	≥260	271.0	T 0611-2011

Note: Technical requirements follow JTG F40-2004. Penetration (T0604) and softening point (T0606) were tested per JTG 3410-2025, equivalent to ASTM D5 [20] and ASTM D36 [21], respectively.

**Table 2:** Physical properties of SBS modified asphalt.

Test Metrics	Technical Requirements	Test Results	Test Method [18]
Penetration (25 °C, 100 g, 5 s, 0.1 mm)	30~60	44.5	T0604-2011
Ductility (5 cm/min, 5 °C, cm)	≥20	33.8	T0605-2011
Softening point (°C)	≥60	70.7	T0606-2011
Elastic recovery (25 °C, %)	≥75	86.6	T0662-2000
48 h softening point difference (°C)	≤2.5	1.9	T0661-2025

Note: PG 76-22 [22] equivalency is based on standard correlations with softening point (required > 60 °C, measured 70.7 °C) and elastic recovery (required > 75%, measured 86.6%) under Chinese industry specifications (JTG F40-2004).

**Table 3:** Physical properties of rubber modified asphalt.

Test Metrics	Technical Requirements	Test Results	Test Method [18]
Penetration (25 °C, 100 g, 5 s, 0.1 mm)	50~70	56	T0604-2011
Ductility (5 cm/min, 5 °C, cm)	≥15	19	T0605-2011
Softening point (°C)	≥55	73	T0606-2011
Elastic recovery (25 °C, %)	≥60	77	T0662-2000

Note: Compliance verified against CJJ/T 273-2019. The measured softening point (73 °C) is consistent with a PG 70-22 to PG 76-22 grading based on standard specification correlations [22].

**Table 4:** Road performance test design.

Types of Asphalt	Road Performance	Test Methods [18]	Test Procedure
70# asphalt, SBS modified asphalt, rubber modified asphalt	High-temperature stability	Rutting test	T0719-2025
	Low-temperature stability	Low-Temperature bending test	T0715-2025
	Water stability	Freeze–Thaw splitting test	T0729-2025

**Table 5:** Significance analysis of the fitting equation for harmful fume concentrations in three types of asphalt.

Types of Asphalt	Fitting Equation	Variance Source	Type III SS	DF	MS	F	*p*
70# asphalt	NH_3_	Regression	63.192	2	31.596	348.306	0.0002808
		Residual	0.272	3	0.091	/	/
	NO_X_	Regression	617.839	3	205.946	205.045	0.00486
		Residual	2.009	2	1.004	/	/
	SO_2_	Regression	36.800	2	18.400	102.060	0.00174
		Residual	0.541	3	0.180	/	/
	H_2_S	Regression	9.770	2	4.885	124.365	0.0013
		Residual	0.118	3	0.039	/	/
SBS modified asphalt	NH_3_	Regression	75.369	2	37.684	66.030	0.00331
		Residual	1.712	3	0.571	/	/
	NO_X_	Regression	251.938	3	83.979	114.237	0.00869
		Residual	1.470	2	0.735	/	/
	SO_2_	Regression	110.571	2	55.285	173.224	0.0007954
		Residual	0.957	3	0.319	/	/
	H_2_S	Regression	10.135	2	5.068	56.857	0.00412
		Residual	0.2674	3	0.089	/	/
Rubber modified asphalt	NH_3_	Regression	269.112	3	89.704	364.051	0.00274
		Residual	0.49281	2	0.246	/	/
	NO_X_	Regression	812.841	3	270.947	177.665	0.0056
		Residual	3.050	2	1.525	/	/
	SO_2_	Regression	90.370	2	45.185	72.288	0.0029
		Residual	1.875	3	0.625	/	/
	H_2_S	Regression	42.445	2	21.222	239.552	0.0004909
		Residual	0.26578	3	0.089	/	/

**Table 6:** Optimal inhibitor content and maximum overall satisfaction for three types of asphalt obtained through satisfaction function optimization.

Types of Asphalt	Optimal Inhibitor Content, x* (%)	Maximum Overall Satisfaction, $D_{Max}$ (%)	Types of Asphalt
70# asphalt	0.082	98.870	70# asphalt
SBS modified asphalt	0.079	98.163	SBS modified asphalt
Rubber modified asphalt	0.080	97.145	Rubber modified asphalt

**Table 7:** Predicted concentrations and degradation rates of each gas at optimal inhibitor content for three types of asphalt.

Types of Asphalt	Predicted Concentration of Each Gas (mg/m^3^)/Degradation Rate (%)			
	NH_3_	NO_x_	SO_2_	H_2_S
70# asphalt	0.229/41.2	0.296/54.0	0.035/77.4	0.086/44.9
SBS modified asphalt	0.239/44.5	0.328/51.7	0.032/83.7	0.086/48.6
Rubber modified asphalt	0.269/43.8	0.322/55.8	0.032/81.7	0.090/52.1

**Table 8:** Test results of road performance for asphalt mixture at optimal admixture content.

Types of Asphalt	Inhibitor Content (%)	Dynamic Stability *DS* (Times/mm)	Freeze–Thaw Crack Resistance Ratio ${TSR}_{f}$ (%)	Maximum Bending Tensile Strain $\varepsilon_{B}$ (με)
70# asphalt	0	2103	78.73	2321
	0.082	2043	79.51	2398
SBS modified asphalt	0	4287	86.13	2760
	0.079	4166	87.01	2850
Rubber modified asphalt	0	4788	89.22	2821
	0.080	4670	90.19	2909

## Data Availability

The original contributions presented in this study are included in the article. Further inquiries can be directed to the corresponding authors.
